# Microscope and spectacle: On the complexities of using new visual technologies to communicate about wildlife conservation

**DOI:** 10.1007/s13280-015-0715-z

**Published:** 2015-10-27

**Authors:** Audrey Verma, René van der Wal, Anke Fischer

**Affiliations:** School of Biological Sciences, University of Aberdeen, Auris, 23 St. Machar Drive, Aberdeen, AB24 3UU UK; School of Biological Sciences, Aberdeen Centre for Environmental Sustainability (ACES), University of Aberdeen, Auris, 23 St. Machar Drive, Aberdeen, AB24 3UU UK; Social, Economic and Geographical Sciences Group, James Hutton Institute, Craigiebuckler, Aberdeen, AB15 8QH Scotland, UK

**Keywords:** Public engagement, Environmental communication, Visual technology, Wildlife conservation

## Abstract

Wildlife conservation-related organisations increasingly employ new visual technologies in their science communication and public engagement efforts. Here, we examine the use of such technologies for wildlife conservation campaigns. We obtained empirical data from four UK-based organisations through semi-structured interviews and participant observation. Visual technologies were used to provide the knowledge and generate the emotional responses perceived by organisations as being necessary for motivating a sense of caring about wildlife. We term these two aspects ‘microscope’ and ‘spectacle’, metaphorical concepts denoting the duality through which these technologies speak to both the cognitive and the emotional. As conservation relies on public support, organisations have to be seen to deliver information that is not only sufficiently detailed and scientifically credible but also spectacular enough to capture public interest. Our investigation showed that balancing science and entertainment is a difficult undertaking for wildlife-related organisations as there are perceived risks of contriving experiences of nature and obscuring conservation aims.

## Introduction

Wildlife conservation-related organisations are key social actors in communicating matters of concern surrounding wildlife loss. Conscious of their own reliance on public support and associated challenges, these organisations are well-versed in crafting public outreach and awareness-raising activities. These range from unidirectional educational campaigns and advertising and branding projects, to citizen science research with varying degrees of public participation, the implementation of interactive media strategies, and the expansion of modes of interpretation to include digital platforms.

Such efforts are located within a context of continued environmental degradation, leading to concerns that there might be communication ‘failures’ between researchers, policy-makers, the media, conservation practitioners, and the general public (Sunderland et al. 2009; Kahan 2010; Bickford et al. 2012). These concerns have, in turn, resulted in the ‘increasing prominence and consolidation of environmental communication’ (Hansen 2011, p. 9) as a pragmatic and constitutive discipline (Cox 2013). Environmental communication research is based on the idea that an issue such as species loss is a ‘matter of concern’ rather than simply a ‘matter of fact’ (concepts coined by Latour 2004, 2008), and that the will and the means for the conservation task is necessarily supplied by an eco-literate and engaged public (Jacobson 1999; Novacek 2008). In short, environmental communication efforts are wrapped up in the question of what might make people care for nature (Milton 2002).

Consequently, communication and engagement projects have placed new demands on organisational resources, influenced strategic practices, and created new roles within conservation organisations. Such changes are an expression of conservationists’ endeavours to educate, enrol, engage with, and keep people within the conservation fold, and to garner the social, political, and economic momentum needed to address large-scale matters of concern. Additionally, these campaigns indicate the socio-economic context within which modern conservation organisations operate (Büscher et al. 2012). The need for public awareness and support is, therefore, not merely an end in itself. ‘Marketing’ strategies validate the existence of a given organisation, keeping it in business, often through literal financial means such as entrance fees from visitors, donations and membership fees (Kitchin 2004).

New visual technologies are now recognised as being a vital part of the communication and education repertoire employed in the conservation world (Clements et al. 2007; Cox 2013). These media, such as closed circuit television set-ups (CCTVs), web cameras, trail cameras, image-based mobile technology applications, and satellite imaging, have been adapted by ecologists and technologists for biodiversity research and monitoring. The modified-for-purpose visual technological vehicles are now also widely used for public engagement, allowing users ever-more intimate views of nature through remote electronic means. These technologies afford viewers the benefits of greater immediacy, increased magnification, higher resolution, night vision, longer battery life, and expanded possibilities for interactivity with e.g. remote control facilities.

While research on environmental communication has traditionally focussed on analyses of ‘textual, rhetorical and linguistic construction’ (Dobrin and Morey 2009; Hansen and Machin 2013), visual imagery and image-making has more recently become a subject of interest. This is not least because of observations that communication in Western contexts is heavily image-based (Jenks 1995) and that the public ‘vocabulary’ of the environment is largely constituted of visual images (Hansen and Machin 2013). This has been reflected, for example, in the ongoing scholarly conversation about the role of visualisation, visibility, and sight in the communication and perceptions of climate change (Rudiak-Gould 2013). Within existing visual environmental communication, studies have primarily focused on the products of communication (images) and impacts (effects on the public), and the relations between the two. However, there have been fewer critical analyses regarding the production processes by which key organisations construct and present nature to the public in the first instance (Christophers 2006; Doyle 2007). In the case of biodiversity conservation communication, critical and relevant insights may be drawn from examinations of natural history photography, programming, and filmmaking (Bouse 2000; Blewitt 2010, 2011; Mitman 2012). In tracing the conflicting interests and configurations of power involved in producing knowledge about wildlife through images, these studies have recurrently observed tensions between ‘simulated spectacle and the objectivity of science’ (Vivanco 2002).

In our qualitative exploration of new visual technologies as used by wildlife-related organisations for public communication, engagement, and education, we offer insight into the production of images by conservation organisations, and examine the logic behind image-making for these institutions. We identify the functions of these technologies for the wildlife conservation cause, and explore the controversies emerging from the multiplicity of these functions.

### Theoretical background: Cognition and emotion

At a general level, conservation practitioners, policy-makers, and educators have recognised the need for both emotion and cognition (i.e. knowledge) to inspire environmental interest, awareness, caring, and even love to motivate pro-conservation behaviour (Iozzi 1989; Kals et al. 1999; Hinds and Sparks 2008; Novacek 2008; Wilson 2008; Earthwatch Institute 2013). Historically, there have been a variety of perspectives on the relationship between emotion and cognition. Barbalet (1998; see also Milton 2002) discusses three perspectives: (i) that emotion is opposed to and distorts reason; (ii) that emotions support reason; and (iii) that emotions constitute rational thought. While reason is a term that has traditionally been used in philosophy and continues to be used in sociology and anthropology in semantic opposition to emotion, the underlying construct of interest here would, in modern psychological terms, best be called cognition. Emotions, in turn, have also been conceptualised as being partly dependent on cognition, since affective states only become meaningful through cognitive interpretation (Niedenthal et al. 2006). Critically, these perspectives underscore that the relationship between emotion and cognition is conceptually complex, and there is little consensus about interactions and direction of causality.

While an in-depth exploration of the precise relationship between emotion and cognition is beyond the scope of this paper, we note that more recent strands of research and theory in the area support the view that emotion plays an intrinsic role in cognition, and that affect and knowing are intertwined (Lazarus et al. 1984; Damasio 1994). These findings therefore add another dimension to the much-espoused conservation ideology that knowing is caring. They hold the implication of emotion being a key component of knowing, as much as knowing is a key element of feeling. This added dimension is reflected in heightened calls to acknowledge the role of emotions in human–wildlife interactions (Manfredo 2008; Jacobs et al. 2012), and to examine the importance of emotional connections between humans and non-human animals for encouraging a sense of caring for nature (Vining 2003).

In environmental education, the oppositional relationship between emotion and reason appears to be part of what Littledyke (2008) summarises as being a continued privileging of the ‘modernist’ model over a ‘constructive postmodern’ stance. The former, which is characterised as being ‘reductionist, determinist, mechanistic and value-free’, is juxtaposed against the latter, which acknowledges the social and affective features of science and science education. Littledyke thus suggests that the effective transmission and receipt of knowledge requires both cognitive and emotional elements. In terms of nature conservation, Milton (2002) firmly reinserts emotions into the conservation discussion with her argument that emotions are a basic mechanism by which humans connect to the environment, and is consequently concerned with the devaluing of emotions and embodied experiences in favour of cognitive ways of relating to nature.

Although reason and feeling are intertwined in important and complex ways, there remains both a theoretical case for and cultural persistence of the separation between affect and reason (Barbalet 1998; Manfredo 2008). While the cultural opposition between knowing and feeling has larger consequences, particularly in terms of the devaluation of the latter (Manfredo 2008), an analytical separation may be considered as a heuristic device. This is not least because the terms are used intuitively and in everyday terms as separate concepts, and this distinction is in many cases pragmatically useful. Although we do not lose sight of the aforementioned enmeshment (see “Discussion” section), and finer distinctions are made elsewhere in the literature (Wilson 2008), we associate here feelings with affect or emotions, and knowing with reason or cognition.

While cognition and emotion were not a priori concepts with which we designed our study, we found these to be pertinent in our data. The following sections of our paper will unfold how new visual technologies were employed by our case study organisations to appeal to both the affective and cognitive needs of audiences. Through an empirical analysis of four initiatives employing new technologies for communicating biodiversity issues, we observe that the ‘uneasy and conflicted alliance’ between the ‘mechanical reproduction’ of wildlife as images, and wildlife conservation (Springer 2012) is in no small part due to the ambiguities and tensions between emotion and reason.

## Materials and methods

### General approach

Fieldwork, interviews, and archival research were undertaken with several key wildlife conservation-related organisations in the UK between January 2013 and May 2014. Since the constantly shifting nature of technology means that a technical definition of new digital technologies would not have been useful in determining a sample population, we looked instead at the range of technologies that were being used by conservation organisations. Almost all of the largest nature conservation organisations use, to some degree, new digital technologies for the production and public dissemination of information to do with their causes. These communication-enabling technologies were mostly visual, or contained heavily visual elements. We strived from the outset to study diverse uses of technology. The cases chosen here therefore represent a range of technologies (cameras, live streaming, tagging and satellite tracking, and mobile applications) that have been used by conservation organisations over the past decade. The cases also provide a diversity of organisational affiliations (government bodies, research-based organisations, commercial, and/or charitable outfits) and a variety of use contexts (physical visitor centres, online facilities and websites, and mobile applications).

### Data collection

Four case studies provide the basis for our analysis, and these include the use of cameras at Huntly Peregrine Wild Watch (run by Forestry Commission Scotland) and the Scottish Seabird Centre; the use of tracking and mapping facilities in the Tollie red kites project run by the Royal Society for the Protection of Birds; and the Zoological Society of London’s use of the camera-trap-image-based application, Instant Wild. Semi-structured and unstructured interviews were undertaken with a range of participants who worked on the technological projects at each organisation (see details below). Interviews focused on these visual technological projects, and were aimed at understanding the social and practical dimensions of technology use. Key questions therefore revolved around clarifying the technological instruments in use, how these were used by the organisations, what the purposes of such technologies were, and how these helped organisations to achieve their stated goals. They further probed the associated challenges and limitations, technological changes over time, and values and opinions in relation to the use of these technologies.

Interviews were recorded and transcribed verbatim. These transcripts, alongside our notes from participant observation fieldwork, formed our primary data. Secondary data sources include public domain text (such as information from websites, press coverage of projects, and user input in the form of forum board comments and reviews). These data sources were selected based on relevance to our focus on the chosen technological projects.

We drew on interviews, field notes, and secondary texts to understand the logic and practices underpinning the use of new visual technologies, as well as to establish the general organisational background and context. This context formed the basis of the brief case descriptions offered below.

### Case studies

Four cases provided the data for the analysis presented here:

(1) The Tollie red kites project run by the Royal Society for the Protection of Birds (RSPB) has its history in the reintroduction of red kites to the UK between the late 1980s and mid-90s. While numbers of this charismatic bird of prey have steadily increased in the England and the reintroduction has been deemed a success, red kite numbers remain low in Scotland. This has been partly due to raptor persecution. A tagging project was started in 2009 to determine where and how birds were being persecuted. These tagging efforts were accompanied by a larger public outreach campaign (initially known as Eyes to the Skies) that included a public website with an interactive map that showed locations visited by kites, based on satellite tag data (Van der Wal et al. 2015). RSPB project officers also used these maps in classroom settings and at community events. A visitor centre and feeding station where the public are able to view red kites in person also exists in Tollie, Dingwall, Scotland. Key respondents for this case included three RSPB officers involved in the project, a member of the organisation’s education team, and three website developers.

(2) Instant Wild is a multi-purpose (surveillance, monitoring, and communication) project created by the Zoological Society of London (ZSL), driven by advancements in the use of Global Systems for Mobile communications (GSM) technology for camera traps. In its main current form, Instant Wild is an application available as both a website facility and a downloadable application for mobile devices. This application is a citizen science effort to crowd-source identifications on wildlife images caught on ZSL camera traps across the world. Images are sent to users’ mobile devices for identification, and users are ranked based on speed and number of identifications contributed. As at April 2014, the project had been in a trial phase for 2 years to determine the feasibility of crowd sourcing image identifications to aid processing large volumes of biodiversity data. By this time, the application had over 150 000 downloads and approximately 1.3 million identifications on over 4600 images. Future plans for the wider project include setting up a grid of cameras for planet-wide biodiversity monitoring and anti-poaching surveillance. Key respondents included two technical advisors and the Instant Wild app and website developer. Notes from the Instant Wild symposium (2014) and website/app also constitute data.

(3) Huntly Peregrine Wild Watch (Peregrine Watch) was, until 2013, a visitor centre located in the Bin Forest in Huntly, Aberdeenshire. The project began in 2003, at a time when use of close-circuit televisions trained on wildlife was becoming popular across UK, and as an extension to surveillance measures implemented in response to raptor persecution. The Bin Quarry has been home to peregrines since 1985. Peregrine Watch was intended as a short-term project under the Forestry Commission Scotland’s (FCS) remit, for outreach and awareness-raising. The visitor hides were designed as a starting point to encourage visitors to explore the forest. Annual visitor numbers varied between 1200 and 8000 over the decade. Later into the project, stills taken at five-second intervals from the primary cameras were broadcast on the FCS website. Key respondents here include the site warden, the former district manager of the Huntly area, two Radio and Electronics Branch technical officers (one retired), a tourism development manager, and an interpretation officer (all from FCS).

(4) Opened in 2000, the Scottish Seabird Centre (Seabird Centre) is a nature-based visitor attraction designed around the use of remote camera technologies. The site is located on the North Berwick harbour, overlooking the Firth of Forth. Through the use of sixteen camera feeds broadcast interchangeably over nine projectors, views from the nearby Dunbar Harbour, and the islands of the Firth (Craigleith, Isle of May, Fidra, and Bass Rock) are made remotely accessible to viewers. Paying visitors are given access to the Discovery Centre, where they are able to control live cameras using joysticks. This control affords two viewing customisation options: a 360° sweep across the panorama, and zoom in/out of the given images, giving close-up views (of up to 30× magnification). Streams from cameras at the Centre are also available for limited viewing on the SSC website. The Centre receives approximately 250 000 visitors annually. Key respondents include the founder of Centre, its chief executive, two operations managers for the Discovery Centre, members of the operations team, three science communications and education personnel, two boat guides, and four long-time volunteers.

**Fig. 1:**
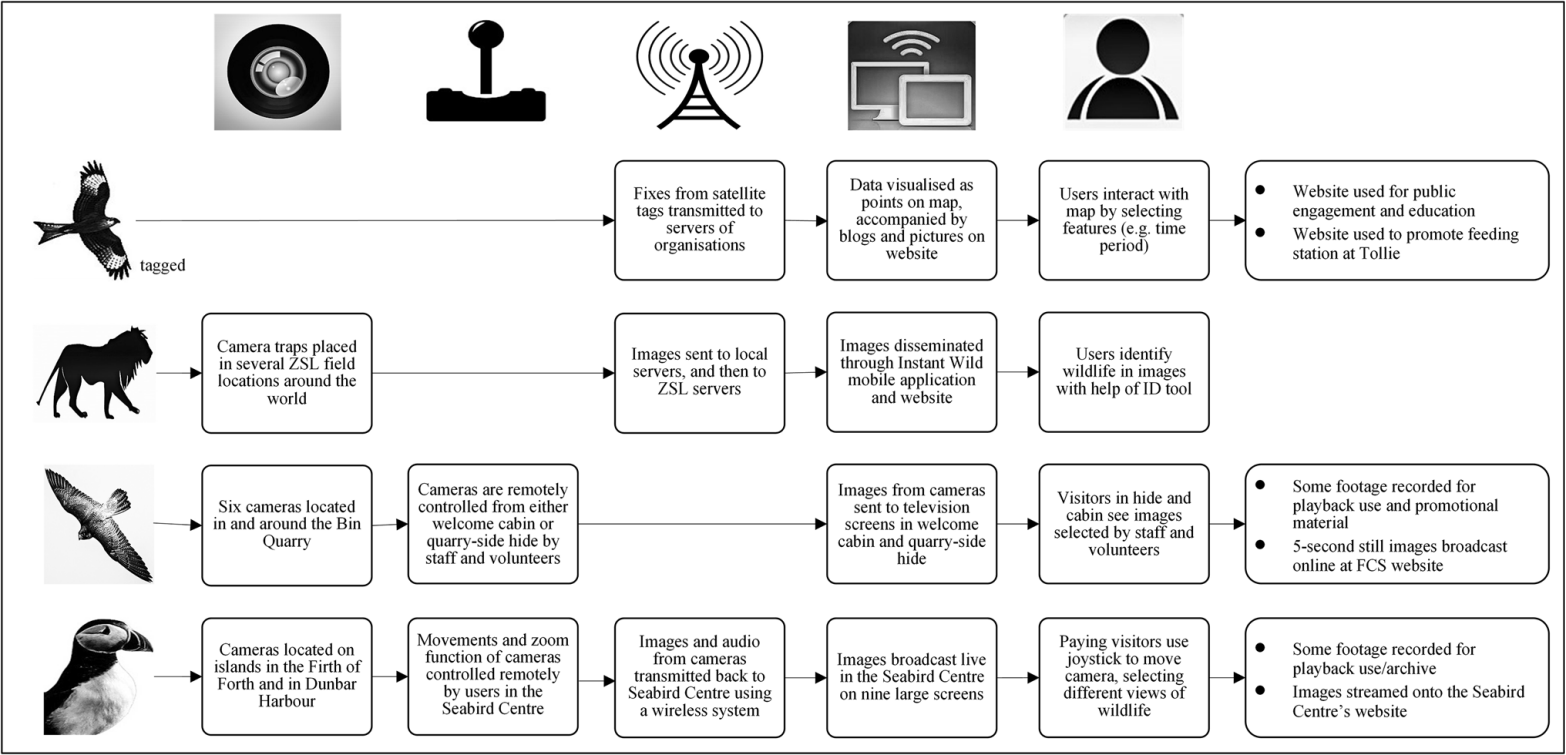
How technologies mediate human–nature relations in our case studies. On the *top horizontal line*, the five icons represent, from *left* to *right*: *1* image-making technologies used, *2* how these devices were controlled, *3* data transmission methods, *4* how data was visualised and disseminated, *5* how users or visitors received these images, and *6* other ways in which images were used. On the first vertical column, the four icons represent, from *top* to *bottom*: the Tollie red kites tracking project, Instant Wild, Huntly Peregrine Wild Watch, and the Seabird Centre

**Fig. 2:**
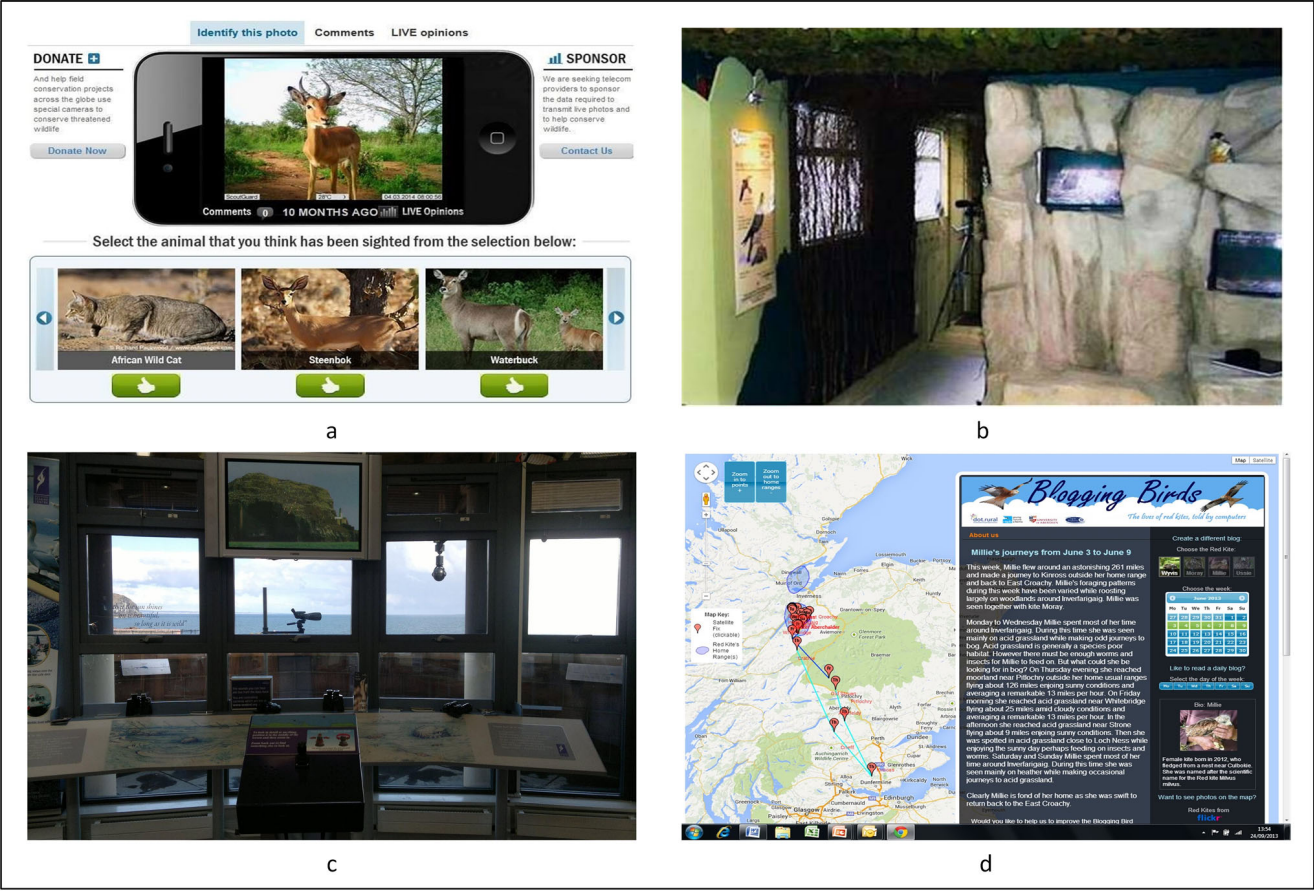
User interfaces for connecting to nature. Clockwise from *top left* **a** screenshot from Zoological Society of London’s Instant Wild application (Source: Zoological Society of London), **b** cabin at Huntly Peregrine Wild Watch, **c** viewing deck at the Scottish Seabird Centre, and **d** screenshot of Red Kites tracking project

A summary of how the focal technologies in these case studies mediate human–nature relations is presented in Fig. 1. Figure 2 depicts the user interfaces from each of our cases.

### Data analysis

We started our analysis in a grounded manner with an exploration of the data, reading through the transcribed interviews to identify recurring themes. A key theme emerging from the interviews was the function of technology. It was constituted by text that referred to the different functions of a technology, including education, awareness-raising, interpretation, attracting visitors, sparking interest, and creating emotional and/or community ties. Using these sub-categories as a coding framework, we then coded all text associated to one or more of these functions in the full set of transcribed interviews, field notes, and additional material.

During a further analytical step, it became apparent that all of these functions fell into one or both of two large groups, which we metaphorically call here ‘spectacle’ and ‘microscope.’ These abstract umbrella themes capture and articulate the running duality of cognitive and emotive aspects of technological wildlife conservation campaigns. These labels were inspired both by the literature and our data: Following the influential work of theorist Guy DeBord (1983), the critical term ‘spectacle’ has been featured in literature examining the representation of nature as images (Dobrin and Morey 2009; Igoe 2010). The term ‘spectacle’ was also used by some of our respondents to describe certain visual arrangements and events designed to evoke enthrallment (for example, newer hides designed to offer panoramic views that privilege visuals over experiences involving sounds, smells, and other senses). The concept of microscope emerged in an interview with an executive staff member of one of our case sites, who actively used the term to describe the opposite of spectacle.

We emphasise here that ‘microscope’ and ‘spectacle’ serve as data-derived devices that metaphorically express the cognitive and affective functions fulfilled by featured visual technologies and resultant images. Just as reason and emotion overlap in complex ways, so do the functions of ‘microscope’ intertwine with that of the ‘spectacle.’ While we maintain a pragmatic separation for the purposes of this paper to reflect the data provided by our respondents, we offer a critical examination of the complex interplay between microscope and spectacle in the discussion section.

## Results

### Microscope: Using new visual media to facilitate knowing

In our case studies, technologically-driven campaigns were often couched as ‘hearts and minds’ projects, representing attempts to simultaneously deliver science-derived facts and to evoke supportive emotional responses for conservation causes. In our interview with an executive staff member instrumental in the founding of the Seabird Centre, our respondent explained that his vision in using remote viewing camera technologies was to provide a ‘microscope’ that would educate the public about the seabirds on the islands of the Firth of Forth. This seemed to apply across all four studied organisations: The capacity for magnification and remote observation via cameras and data visualisations across our case studies meant that the technologies in question acted as a metaphorical ‘microscope’ in revealing details about wildlife, and were intentionally used for this purpose. We observed that alongside traditional media, these technologies allowed practitioners to frame, guide, direct, and inform the vision of members of the public. Through the technological microscopic lens, organisations inducted visitors and users to particular, cognitive ways of observing, understanding, and relating to nature achieved through remote observation focused on (i) behaviour, (ii) morphology, (iii) identification, and (iv) monitoring.

The visual technologies studied by us exposed *behaviour* that would otherwise have been impossible to see, or could previously only be seen by dedicated or lucky enthusiasts. These visual media were presented partly as an invitation for the public to observe otherwise hidden behaviours, such as movement routes, as with RSPB’s Eyes to the Skies. Using interactive maps visualising red kite movements based on satellite-tracked data, users could select particular birds, time periods, and/or geographical areas to follow the movements of the tagged kites.

“[On the website, there was a] *maps section and you could click on a particular red kite. It was different levels of detail on that*. [Users] *could look at* [the kites’] *daily adventures or its weekly adventures, or every single adventure it’s had since it fledged* [… Users] *could see times, and could get some idea of speed of flight as well, by looking at distance covered, and looking at what times that was between. So you could get quite a bit of information about the birds*.” (Red Kites operations)

Some of the footage and visual data revealed information about the behaviour of a species that was previously unknown or little known by the public, and indeed sometimes even by practitioners. In the case of Peregrine Watch, a former member of the Forestry Commission of Scotland’s managerial staff explained in some length that visitors, practitioners, and experts involved in Peregrine Watch learned a lot from observation mediated by cameras. Particularly surprising to them was the complex social interactions between peregrine falcons, which even the advising experts for the site (a local raptor specialist interest group) did not anticipate:

“*And I think, just for interest’s sake, we felt that a camera, especially if it was recording the actions of the bird, would give us an insight into the habits and lifestyle of the birds. And certainly, that was one of the big successes of the project, was our understanding of the birds, the complexity of their social lives was a way beyond anything that we had even dreamt about*.” (Peregrine Watch managerial 1)

Another set of social behaviours that these technologies exposed to the public was courtship, mating, and nesting rituals. With Peregrine Watch, one of the main cameras on site used for public broadcast and live website streaming was an infrared camera on a known nest site (eyrie) on the quarry face. According to the warden of the site, this camera proved particularly popular and useful for exposing otherwise hidden peregrine-chick interactions:

“*The infrared camera on the eyrie with the sound, because the grass grew, the public couldn’t see the nest, they couldn’t see eggs or chicks, until they were mobile at about two weeks. And that’s where this monitor behind was linked to that camera and that was the reason behind it. So that the public could see what was happening behind the grass* […]. *And the infrared overnight has been excellent ‘cause it’s given us and the public a view of what peregrines do at night. We were doing this long before BBC Wildlife and SpringWatch were doing it*.” (Peregrine Watch operations)

Live streaming meant that viewers could see unedited footage and were exposed to mundane reality rather than eventful action. However, staff on site did focus on frames with most potential for observing easily interpretable behaviour. At the Seabird Centre, during gannet mating season, cameras were often pre-set, and staff guided visitors to bring back into frame paired birds (which tended to stay in the same locations). Part of the reason for highlighting paired birds was to allow visitors to learn and begin to recognise unique, predictable, and consistent behaviour, such as gannet courtship rituals of beak fencing and sky-pointing. They also used recorded ‘highlights’ fairly frequently during interactions with visitors. As explained below, such pre-recorded footage was arguably better for educating the public, as the images became an available source for staff to accompany delivery of an expert interpretation of the nature on show.

"*…winter time, from a wildlife point of view, most of the seabirds aren’t around, so things like the guillemots, the razorbills, the gannets, the puffins, the kittiwakes, they’re not really on camera anyway. So there’s the argument to say, well it’s actually better for our visitors to show them recordings of the previous season* […]. *And it’s actually a lot more they can gain from watching that and having that interpreted for them, then by moving a live camera around on an island where there isn’t a huge amount to see anyway*.” (Seabird Centre operations 1)

Apart from enabling a focus on behaviour, visual technologies were frequently used—in tandem with traditional modes of interpretation—to familiarise the public with *morphology*. At the Seabird Centre, for example, one of the features heavily advertised to draw visitors in was the interactive aspect of the live camera set-ups. The zoom function allowed visitors and staff to magnify, as one might do with a microscope, visible morphological traits. This was similar to Peregrine Watch, where a member of the operations staff explained that she had used the cameras to create an in-depth, direct educational experience centred on identifying features and behaviour of peregrine falcons:

“*Now, the quarry face* [camera] *was brilliant because you could zoom in and show people and this was what we were able to do at the bottom when we got the technology with the control panels. We were able to zoom in and show the public the talons, the beaks, how they were able to pluck food and you’re kinda working with them, using the camera equipment and what you were seeing as a direct experience*.” (Peregrine Watch operations)

This zoom function also inducted viewers into the task of *identification*. In the case of the Seabird Centre, visitors were invited to observe morphological detail to differentiate between similar seabirds such as razorbills and guillemots. With the Instant Wild application, users identified animals captured in a given image by selecting from a list of species that were likely to be caught by that particular camera. However, this was not as simple a task as it first appeared. Instant Wild’s camera often captured images of similar-looking species (for example, of the numerous species within the antelope group, on the Kenya cameras), or, due to technical limitations, blurred or partial images of smaller or fast-moving species. Making a positive identification, therefore, required informed and skilled vision on the part of users.

As a consequence of the focus on identification, the visual technologies we studied were also connected to biodiversity *monitoring* efforts that involved members of the public. This happened on a localised scale with log books that kept track of wildlife sightings at and around Peregrine Watch and the Seabird Centre. It also took the form of more ambitious projects such as Instant Wild, which crowd-sourced identifications on larger quantities of imagery, with the intention of scaling up to obtain species occurrence data over time. Monitoring efforts also turned up elusive species, which would have otherwise been difficult to track due to remoteness of terrain, nocturnality, or rarity. With Instant Wild, while most of the images captured by the camera traps for public identification were of common species, the set-up had captured images of a scarcely recorded mountain mouse deer (on its Sri Lankan camera) and a critically endangered Javan leopard (on its Indonesia camera), thereby confirming the existence of these animals in those locations.

The visual technologies in our case study projects did not only go some way in making behaviour, morphology, and numbers of non-human nature apparent. Organisations also boasted that these technologies afforded knowledge and insight remotely, without human ‘intrusion’ and the potential of damage to wildlife arising from any direct, unmediated contact between people and nature. Our interviewees reasoned that non-intrusive technological viewing through cameras, images, and data visualisations constituted unaltered access to ‘raw nature’ i.e. observing ‘real’ animal behaviour without observer effect. This was partly a direct response to the original intentions behind the implementation of several campaigns, where technologies were used as a crime prevention measure (i.e. to detect poaching, persecution, and egg theft, as was the case for Peregrine Watch and the red kites tracking project). Non-intrusive observation was also considered a selling point by our case study organisations, and this was seen in online and marketing material, where potential visitors were told that electronic viewing would afford live close-ups without disturbance to the wildlife.

“*You don’t want to disturb the wildlife. So I just thought it’d just be ideal. Particularly, we’re near the city, so you could get the kids out, they could see wildlife without doing any damage to the wildlife itself, you know*.” (Peregrine Watch technical 1)

“*And also, there’s the argument that *[…] *by viewing the birds through the cameras, you’re actually observing them more in their natural environment, than if you were stood several metres away, peering at them through binoculars, you know, because the birds do not notice the cameras at all. They just carry on life completely oblivious to our equipment out there, so what you’re actually observing is raw nature, and* […] *there’s not even any human intervention to make the birds behave any differently*.” (Seabird Centre operations 2)

### Spectacle: Using new visual media to facilitate feeling

“*…there’s no underlying message *[… Not] *every visitor must know that there’s a 150 000 gannets on Bass Rock or that puffin numbers are in decline, or that there’s too much plastic in the ocean that’s killing wildlife. We don’t have anything set in stone in that sense. What we want is for* [visitors] *to go away feeling very enthused about the wildlife that we had on our cameras here, and the experience that they’ve had* […]. *You need to get them engaged first ‘cause if they’re not engaged, they don’t care about the wildlife, then they’re not going be engaged then with the other messages and so anything else that we’re trying to* [convey].” (Seabird Centre operations 1)

Although considered by the organisations we studied as a key aspect, the uptake of techno-visual instruments in our case studies was rarely purely for producing and disseminating science-based knowledge of the natural world through using these media as microscope. Rather, as our respondent above indicated, organisations also undertook image-making and used images with the intention of getting as many members of the public as possible ‘engaged’ and caring for issues that were removed from their day-to-day experiences. The same technologies and images used to fulfil cognitive functions were also used in the creation of a metaphorical ‘spectacle’—“incredible close-up” images and visual experiences designed to capture interest, to the end of creating a necessary initial emotional, normative ‘connection’ with members of the public. This required nature to be (i) accessible and novel, (ii) emotionalised, and (iii) personified.

At one level, new visual technologies were used by our case study organisations to bolster *access* to the natural world and the spectacle therein. Technologies such as mobile applications and cameras were viewed by respondents as means of facilitating social inclusion, of drawing in and disseminating information to people who may have wanted to but were physically unable to access nature in person, either due to distance or inability/disability:

“*And part of it was, as I said before, to get pictures from here down to the bottom for people who weren’t able to come up themselves, you know. For the disabled or less able to walk up themselves*.”(Peregrine Watch technical 2)

“*I think it’s really an incredible thing for people to be on the website, to be on the iPhone out there sitting at their whatever job they’re doing, and they get a text message or you know, a notification of an elephant in Tsavo has just triggered the camera. And it’s just a way to get people connected with nature, and a way that, you know, there’s nothing else out there like there* [….] *what we’re doing* [gives a] *real time kind of excitement of being able to see wildlife in areas where people might never be able to go to, or might never see that wildlife. So it’s a pretty cool way to get people connected you know to what we’re doing in the field and the species that we’re trying to conserve*.” (Instant Wild operations staff)

Implicit even within the above quotes was a concern beyond access to nature in the interest of inclusivity. Our respondents recognised the need for organisations to improve the *accessibility of nature* in order to encourage the public to ‘connect’ with nature. To interest members of the public who were not already enrolled into the cause, as well as to garner repeat visits, visual technologies were employed as a strategy to make wildlife less remote, detached, or ‘outside’ of people’s day-to-day experiences. For audiences who were more familiar with technology than wildlife, organisations used image-based functionalities to seduce viewers and invoke a sense of fascination and ‘discovery’ with regard to the nature displayed. Additionally, the technologies themselves provided a point of *novelty*, enabling new, and for some, exciting ways of viewing and imagining nature. Both image and image-making thus offered a means by which non-human nature could become accessible on demand and without requiring prior knowledge.

“*Ultimately, what we want are visitors to walk away from the Centre having had a fantastic day out, and a really good experience. Now, if they walk out of there having not gained any new understanding about wildlife, about nature, yet they’ve had a fantastic day, they’ve learnt about how our cameras work, fine, brilliant. They’re gonna go home, they’re gonna write a really good review on TripAdvisor, you know, we’re gonna get good repeat visits from that* […].* I mean, my argument to that would be how would you engage disadvantaged or generally uninterested person* [without]* having a camera there* […] *what we’re doing here is we’re taking that wonder, say the Bass Rock, and we’re actually making it accessible to as many people as possible*.” (Seabird Centre operations 1)

“*And I’ve had lots of people email me saying, ‘oh it’s amazing to receive these images, it transports me to this other place’. People do seem to get a lot from it. And I like to think at least that that gets people, makes people more enthusiastic about conservation, about saving those species they see in those images. If you’re more connected to something, you care more about it. It’s hard to care about something that’s very, very remote from you and very, very much outside of your experience*.” (Instant Wild technical)

The technologies were also viewed as particularly effective in tracking, capturing, and amplifying ‘reliable’ species that exhibited consistent and predictable behaviours that could be easily viewed. These were seen as being easily translatable into guided viewing experiences, allowing organisations to interpret and mediate images for viewers, especially those who may not be ‘geeks’, by establishing an easy understanding and affective connection within a limited interaction time:

“*For someone that’s not a birdwatcher, it’s* [also]* easier for us to show them what a gannet is or what a puffin is, the big, easily identifiable birds. When you get into the realm of waders, because they’re a lot smaller, because they share lot of similar characteristics, it becomes a lot more difficult to explain to a visitor a certain type of wader. It’s not impossible. It’s just more time consuming, more difficult and ultimately, we found that visitors that don’t get as much enjoyment out of those types of birds. There’re not charismatic enough, not predictable is what I think I would say. You know that if you point a camera on a gannet, at some point it’s gonna beak-fence, it’s gonna do some bowing, it’s gonna do some sky-pointing. All these are very interesting things. They’re easy to spot, from a visitor’s perspective, and even from someone who’s not a birdie, who’s not a birdwatcher at all, they can understand.*” (Seabird Centre operations 1)

What the above respondent also highlighted is that accessibility relied on charisma. While the concept of charisma is a subjective one and visual technologies have the capacity to make even the mundane extraordinary by offering unique perspectives, we observed that organisations actively selected charismatic species described as possessing ‘wow factors’ as flagships for technological projects. Although the physical locations connected to the technologies we studied were rich in species biodiversity, focal species were ones that were most easily recognisable, predictable, detectable, distinctive, larger, and yet unique (Lorimer 2007)—species that organisations believed the public found most interesting, and that would provide the most evocative viewing experiences. With the Seabird Centre, despite being located in the naturally abundant Firth of Forth, we observed that live cameras were most frequently trained on puffins (with distinct colourful beaks during breeding season between mid-April to early June), gannets (which gather in the tens of thousands on Bass Rock during breeding and nesting season between late January and October), and seals (which breed mainly on the Isle of May in November and December). With Peregrine Watch, the site was named after and revolved around what was perceived to be the Bin Quarry’s most charismatic species, the falcons, despite a rich variety of species living in the surrounding Bin Forest. Although there was debate over the decision to go with ‘Peregrine Wild Watch’ rather than simply ‘Wild Watch’, our respondents explained that the decision was made partly because the organisation believed that the prospect of watching these raptors, known for reaching high speeds when diving after prey, would draw the public in.

The access(ibility) of wildlife was also a precondition for the production of *emotionalised* images and viewing experiences that elicited affective reactions from observers. Apart from affording greater frequencies of sightings of rare species or visually arresting behaviour and impressive features that visitors and users might not have seen closely, the technologies were viewed as having greater capacity to create intimate emotional experiences, compared to traditional modes of interpretation (such as static information panels). A staff member at Peregrine Watch recalled an incident that she believed would not have been seen and which would not have had an effect if not for the cameras:

“*…on this occasion, the female* [peregrine falcon] *had two chicks, but one chick died. And she spent an afternoon trying to feed a dead chick. She would croon at it and try and get it, to revive it. Now I had the public in, and I had a cabin full of people who spent a couple of hours watching this bird with this dead chick. I had public that were crying, and in the end I had to switch it off* […] *because it was that emotional, that experience. And it still gets me in the throat because in the end, she had to discard the dead chick and then go and look after the living one. So the people that were there related and it was a very emotional thing for them *[…]. *We wouldn’t have seen that if we didn’t have the cameras*.” (Peregrine Watch operations)

Organisations relied on the emotionalised effects of such technologically enabled viewing experiences and images to garner the social and political will of the public and policy-makers. This support was perceived as being important for conservation causes, particularly when faced with issues such as raptor persecution. In the case of the red kite tracking project, the tags, satellite data visualisations in the form of maps, blogs, and the various website facilities were an integral part of a larger approach that

“*…was about connecting the communities around the Black Isle with the red kites, just to try and make people see how bad it was that they were being persecuted* [and] *really, to give us a big platform from which to kind of spread the unfortunate bad news, but that was the only way we could really get people to kind of understand the magnitude of what was going on*.” (Red Kites operations)

Due to the reliability of both charismatic subjects and the visual technologies trained on these animals, there also existed the possibility of mediating a sense of affinity with individual, often named and *personified* animals. With the red kite tracking project, birds were named, and more recent efforts saw each bird having its own blogs and maps visualising its movements. Such features allowed the user community to become acquainted with birds as individuals and lent themselves to press coverage, as was the case with Merida, a tagged female red kite named after the heroine of an animated Disney movie.

**Fig. 3:**
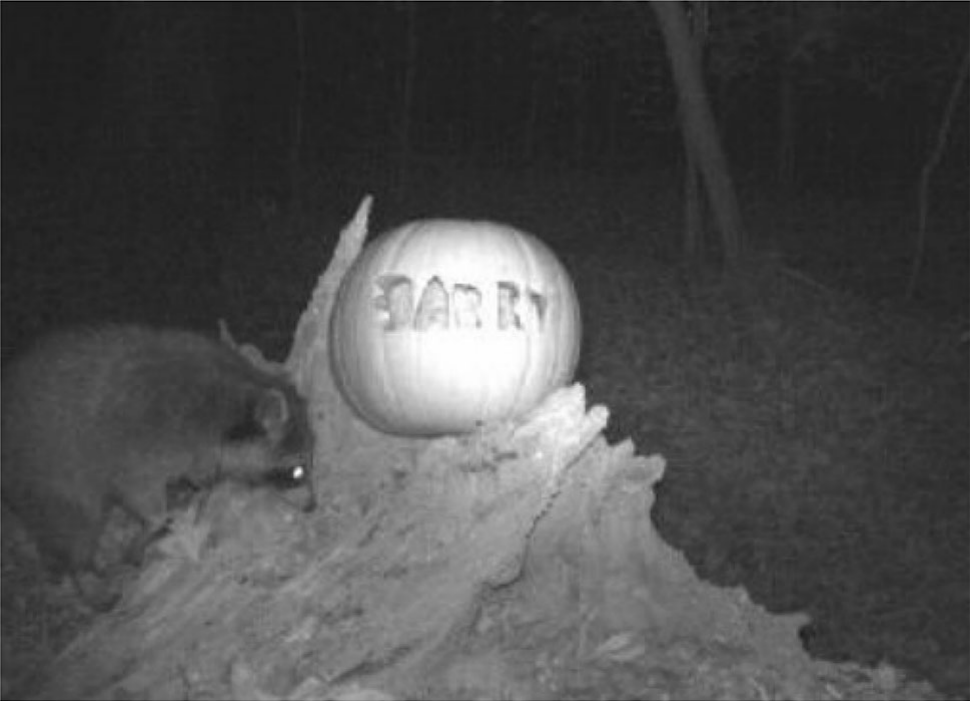
Camera trap-picture of a raccoon taken by an Instant Wild camera in America. Source: Zoological Society of London

Further, the personification of a particular species or individual extends the possibilities for the creation of emotional affinities and communities of interest. With the Instant Wild application, one of the US-based camera traps often caught images of a raccoon that came to capture the imagination of the user community (Fig. 3). Users anthropomorphised the raccoon, and Barry (as the community had named it) garnered a fan following of its own. The personification of Barry created possibilities for Instant Wild to bolster emotional affinities and consolidate the community of interest. In October 2013, for instance, field researchers associated with Instant Wild put a pumpkin carved with Barry’s name out by the camera trap frequented by the raccoon. The resultant images with Barry and the pumpkin generated a higher degree of interaction between users, and between users and researchers, compared to the more usual disjointed user comments on images.

## Discussion

Our analysis of four cases shows that new visual technologies were used by organisations to serve two necessary functions: ‘microscope’ and ‘spectacle’, reflecting, respectively, the cognitive and emotional aspects of public engagement. Given that the nature conservation task relies on public awareness, organisations used technological ‘pedagogies of massification’ (Elliot 2006) to deliver information that was scientifically credible in order to create a knowledge-based public consensus of the objectives of wildlife conservation (Daston and Galison 1992). At the same time, organisations also understood that the information they disseminated had to be spectacular enough to capture and motivate the interest, empathy, and support of the public, toward the fulfilment of organisationally defined conservation objectives.

While our data has shown that organisations considered both knowledge and affective components important for engaging the public, and that new visual technologies were used to fulfil both functions, we have thus far treated the microscope and spectacle as analytically distinct. However, it is apparent that there are, both conceptually and in day-to-day organisational practice, clear functional ambiguities that make disentangling the affective and intellectual functions fulfilled by the technologies difficult. Just as reason and emotion are intertwined in complex ways, we observe that the microscope and the spectacular are interchangeable and fused approaches through which the public can look at nature. Both are used in tandem by organisations toward evoking a sense of caring for wildlife.

The simultaneous featuring of both ‘simulated spectacle’ and the ‘objectivity of science’ (Vivanco 2002) occurred repeatedly within our cases studies, and we offer here three of the more apparent ambiguities. First, through the use of visual technologies, the focus on behaviour was just as easily a privileging of visuals that elicited emotive reactions as an effort to educate. For example, distilled footage of peregrine falcons hunting shown at Peregrine Watch could have been as much a learning experience of the hunting and feeding habits of the falcons, as it was about the spectacular experience of watching a raptor plunge through the sky at high speeds to capture prey. Second, zooming in to show morphological details served the ends of identification as much as it highlighted the spectacular features of a species. In magnifying the morphological details of particular species, it was clear that respondents were also highlighting the visually arresting aspects of these features. Focusing on puffins during breeding season when they have their instantly recognisable colourful beaks allowed the Seabird Centre to show distinctive features identifying the seabird while simultaneously offering a visual understanding that was selective and premised on charisma. Third, where new visual technologies were used to make nature accessible and novel via charisma and personification, organisations often used the same images and techniques as a gambit for educating audiences by supplying accompanying ecological information.

While microscope and spectacle as functions thus overlapped, studies of other visual arrangements for apprehending and disseminating wildlife biodiversity information highlight another salient observation—that there is a ‘constitutive tension’ between the two aspects, primarily as a result of the perceived problems associated with the spectacle (Bouse 2000; Vivanco 2002; Mitman 2012). Following the ideas of critical visual theorists such as Debord (1983) and Baudrillard (1994), the spectacular aspect of image-based representations has been criticised for producing ‘inauthentic’ experiences of nature (Chambers 2007), for promoting ‘irrational’ reactions based on emotions rather than facts (Milton 2002), and for rendering nature a commodity (Brockington and Duffy 2010; Igoe 2010).

These technologically deterministic fears, rooted in the cultural privileging of knowing over feeling, were shared by our case study organisations. It constituted part of the reason for their cautious approach to implementation of technologies, with several respondents lamenting the ‘Springwatch effect’, a term used to describe the situation where visitors and users were seen to be demanding immediately exciting and simulated wildlife spectacles instead of more ‘real’ and mundane experiences of wildlife (see also Blewitt 2010).^1^ In making the distinction between contrived and real nature, the practitioners we spoke to were concerned about the loss of more holistic and direct sensory experiences (involving smells, sounds, and bodily sensations, rather than just sight). They were also concerned about the sensationalising of nature, particularly due to the personification of animals. For example, our interviewees recounted instances where members of the public had ‘become emotional’ and insisted on organisational intervention in situations where wild animals on screen were seen to be in distress. Novel technologies producing greater numbers of ever-more aesthetically evocative and intrusive images of species therefore gave rise to the contention that organisations are creating ‘eco-pornography’, idealised versions of nature (Welling 2009) that may result in fleeting, misinformed, and superficial connectedness to nature.

The spectacular aspects of biodiversity conservation in the form of techno-visual set-ups can also be interpreted as an indication that species loss is becoming a ‘new source of capitalist accumulation’ (Igoe 2010). Moves to stimulate emotional involvement with nature through improved accessibility, personification, and emotionalisation generated concerns about the dilution of wildlife to commodity, packaged for the purposes of eliciting donations, membership monies, and repeat visits. The larger implication here is that spectatorship comes to delineate the extent of public inclusion and participation, in a case of vicarious conservation. Even an interested viewer might find herself relegated to passive supporting roles, with pro-wildlife conservation behaviour limited to supporting organisations via the donate buttons and boxes we found at our case study sites. At the same time, we noted that the use of new visual technologies had the real potential of encouraging critique through spectacle (DeLuca 1999). As a gambit for engagement, the use of these media greatly widened the opportunities and options available to the general public, particularly beginners, for experiencing nature in an accessible way. Further, there was indication that these media opened up avenues for arguably more meaningful public participation in conservation, in the forms of citizen science and volunteering.

## Conclusion

While we agree with Milton’s (2002) view that overly ‘cognitive’ ways of relating to nature ‘serve capitalism well by depersonalising nature’, we have shown that there is little to stem emotions in the conservation realm from being equally susceptible to the problems associated with spectacular visual accumulation. We thus contend that it is neither microscope nor spectacle, and not a given visual technology as such that lends itself to ‘emotional exploitation’ or ‘cognitive depersonalisation.’ Rather, it is the intentions of producers in using these media that ultimately matter, and unpacking the use of these technologies by organisations shows up multiple points of ambiguity and complexity. The balance between microscope and spectacle also emerges from the fact that modern conservation organisations are complex creatures with methods, perspectives, and aims that necessarily evolve alongside the dynamic socio-political and economic landscape (Mace 2014). Our analysis, which has investigated the breadth and ambiguities of the image-making process, thus serves as a call for more nuanced examinations of different technological mechanisms for public interactions with nature, and of the issues surrounding the logic and processes underlying the production of visual representations of wildlife.

## Footnotes

1 Springwatch, Autumnwatch, and Winterwatch are popular annual primetime wildlife programmes run by the British Broadcasting Corporation (BBC). The ‘Springwatch effect’ is a blanket term that refers both to how these programmes appear to rekindle public interest in wildlife, as well as in the sense described here.
